# Developing a decision tool to identify patients with personality disorders in need of highly specialized care

**DOI:** 10.1186/s12888-017-1460-6

**Published:** 2017-08-31

**Authors:** M. Goorden, E. M. C. Willemsen, C. A. M. Bouwmans-Frijters, J. J. V. Busschbach, M. J. Noomx, C. M. van der Feltz-Cornelis, C. A. Uyl-de Groot, L. Hakkaart-van Roijen

**Affiliations:** 1 Erasmus University Rotterdam, Institute for Medical Technology Assessment, Institute of Health Policy & Management, P.O. Box 1738, 3000 DR Rotterdam, The Netherlands; 2 PsyQ, Department of Personality Disorders, The Hague, The Netherlands; 3 Viersprong, Institute for Studies on Personality Disorders (VISPD), Halsteren, The Netherlands; 4 Erasmus Medical Centre, Department of Psychiatry, Rotterdam, The Netherlands; 5 Zaanstad Medical Centre, Department of Psychiatry, Zaandam, The Netherlands; 6 Department of Child Development and Education, University of Amseterdam, Amsterdam, The Netherlands; 7 Department of Tranzo, University of Tilburg, Tilburg, The Netherlands; 8 Clinical Center for Body, Mind and Health, Academic Psychiatry Department, GGZBreburg, Tilburg, The Netherlands

**Keywords:** Personality disorders, Decision tool, Highly specialized care, Validation study

## Abstract

**Background:**

Current guidelines recommend referral to highly specialized care for patients with severe personality disorders. However, criteria for allocation to highly specialized care are not clearly defined. The aim of the present study was to develop a decision tool that can support clinicians to identify patients with a personality disorder in need of highly specialized care.

**Methods:**

Steps taken to develop a decision tool were a literature search, concept mapping, a meeting with experts and a validation study.

**Results:**

The concept mapping method resulted in six criteria for the decision tool. The model used in concept mapping provided a good fit (stress value = 0.30) and reasonable reliability (ρ = 0.49). The bridging values were low, indicating homogeneity. The decision tool was subsequently validated by enrolling 368 patients from seven centers. A multilevel model with a Receiver Operating Characteristic Curve (ROC) was applied. In this way, an easily implementable decision tool with relatively high sensitivity (0.74) and specificity (0.69) was developed.

**Conclusions:**

A decision tool to identify patients with personality disorders for highly specialized care was developed using advanced methods to combine the input of experts with currently available scientific knowledge. The tool appeared to be able to accurately identify this group of patients. Clinicians can use this decision tool to identify patients who are in need of highly specialized treatment.

**Electronic supplementary material:**

The online version of this article (doi:10.1186/s12888-017-1460-6) contains supplementary material, which is available to authorized users.

## Background

The prevalence of personality disorders is high. Several studies have suggested that approximately 1 out of every 10 people in the general population has a personality disorder [1]. When compared to disorders like depression or generalized anxiety disorder, the economic burden is large - this is especially true for the economic costs of borderline and obsessive-compulsive personality disorders [2]. Patients with personality disorders are substantial users of primary care and mental health services [2–6], in particular those with borderline personality disorder. When compared to patients with depression or other personality disorders, they receive the highest amount of care [4]. As a subgroup within this group, patients with severe personality disorders often face additional problems with regards to violence, antisocial behaviour and interpersonal relationships and a greater recurrence of self-harm and a greater duration of administered care [7, 8]. The quality of life of patients who experience severe and complex personality problems in combination with a personality disorder is comparable to adults with depression [9, 10]. As people with personality disorders form a very heterogenous group, the personality disorder diagnosis alone is seldom sufficient for treatment planning [11]. Guidelines advise highly specialized care for patients with more severe personality disorders [12]. This is supported by evidence that indicates that patients with personality disorders are less responsive to usual treatment [9]. However, research concerning the early identification of patients in need of highly specialized treatment is scarce. Therefore in clinical practice, referral to highly specialized care is often only considered after multiple ineffective regular treatments [12]. Thus, patients may receive insufficient and inappropriate treatment [9] and are expected to generate high costs over time.

Referral to highly specialized care may be optimized by improving diagnostics. To date, validated tools for decision support are scarce in psychiatric practice. This is in contrast to other parts of health care, such as oncology or cardiovascular disease. In the absence of validated tools for the identification of patients who may benefit from highly specialized care, clinicians currently rely on overall clinical impressions or severity of symptoms [13].

To develop a validated tool, it is important to first define the characteristics of patients with severe personality disorders. Until now, only a few studies provided definitions of patients with severe personality disorders. Crawford et al. [14] showed that only a few of these studies provide such definitions. These definitions fit five main themes: 1) some categories of personality disorders are more severe than others; 2) severity depends on the number of features of a personality disorder; 3) severity depends on the number of categories of personality disorders; 4) severity depends on the level of impairment in social functioning, and 5) severity depends on the risk of harm towards others [14]. Tyrer [7] and Crawford, Koldobsky, Mulder, and Tyrer [14] developed a severity scale for personality disorders based on the number of clusters, the number of personality disorders, the level of impairment in social functioning and the risk of harm towards each other. However, their scale is not based upon a systematic approach to the evidence. Moreover the relationship between severity, as defined by the criteria of the scale, and treatment allocation to highly specialized treatments is unclear. Although the criteria on this already existing severity scale are expected to partly overlap with the criteria for the identification of patients who may benefit from highly specialized care, these criteria may not cover these patients sufficiently.

As there is no knowledge on how to identify these patients, the aim of the present study was to develop a decision tool that can aid clinicians in identifying patients with personality disorders in need of highly specialized care.

## Methods

### Study design

**Fig. 1:**
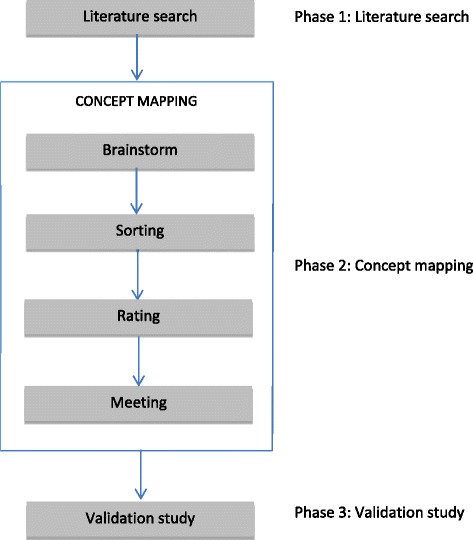
Flow chart describing the methods used in the study

The Decision Tool Personality Disorder (DTPD) was developed by clinicians in collaboration with researchers. Its development progressed through three primary phases. During the first phase, a systematic review of the literature was conducted to serve as a scientific foundation for the decision tool. In this phase, experts were asked to suggest search terms in addition to the search terms that the researchers had already generated. In this way, a large set of potential predictors relevant for treatment allocation was formed. In the second phase, a structured conceptualization methodology known as concept mapping was employed to complement the initial list of features. These criteria were provided by clinical experts and used to develop a consensus-based conceptual framework to guide tool development. Experts were asked to sort the potential predictors into distinguishable categories. In this way, a list of items based on the concept mapping results was generated. These items were used to create the DTPD. Experts were consulted at every step to ensure clinical usability. In the third phase, the instrument was studied for its psychometric properties. An overview of the three phases is presented in Fig. 1.

To effectively take key decisions in the concept mapping process, guidelines recommend to use a small group of participants and/or researchers [15]. Therefore, before the study started a small working group was formed. This group consisted of researchers and clinicians from two mental health care institutions (De Viersprong, a mental health centre specialized in personality pathology; PsyQ, a mental health centre for general psychopathology) and a university (Erasmus University, Institute for Medical Technology Assessment), all specialized in the treatment of personality disorders. This working group contacted experienced clinicians who were then invited to complete a digital survey to provide their contact details and the contact details of other experts for participation in the research.

### Phase 1: Literature search

To develop the first set of criteria for the DTPD, a systematic literature search was conducted in PubMed and Psychinfo. In the absence of studies directly examining factors associated with a need for highly specialized care, proxy indicators had to be identified. The following proxy indicators were defined by experts using a web-based survey: comorbidity, severity, dropout, prognosis and patient characteristics. Search terms were then based on these terms. Studies were first screened by title for selection. Selection was based on eligibility criteria, numbered: (1a) English/Dutch/Human/Abstract or full text available, (1b) Randomized trial/(systematic) Review/Clinical trial/Observational study and (2) published after 1992, (3a) Personality disorder, (3b) Proxy indicators in combination with patient characteristics/comorbidity, and (4) no overlap between studies. For criteria 3b we searched for possible characteristics of patients or certain psychological comorbidities that were associated with having such a proxy indicator - such as certain characteristics that are associated with dropout. Two researchers independently performed the selection process and data extraction of the studies (MG and DS)^1^. Differences in selection were resolved by discussion. Following this, the studies were screened for information on predictors/criteria associated with dropout, severity of the personality disorder, predictors for the course of treatment and other prognostic factors. In the review, we have adopted the Preferred Reporting Items for Systematic Reviews and Meta-Analyses (PRISMA) statement [16]. The criteria defined in the literature search were subsequently used in the concept mapping phase.

### Phase 2: Concept mapping

Concept mapping is a method that integrates qualitative research design with quantitative analytic techniques to conceptualize a phenomenon. The concept mapping in the present study consisted of three successive actions for the participants: a brainstorming session, sorting criteria and rating the relevance of criteria. Participants were given access to an online concept mapping system [17]. The web-based concept mapping procedure consisted of three successive steps:

- The brainstorming session: initial criteria from the review were presented to the panel and subsequently the experts were asked to formulate additional criteria which they thought could distinguish between patients who are in need of highly specialized care and patients who are not in need of highly specialized care. The criteria from the literature review and the additional criteria provided by the experts were merged together and subsequently edited for redundancies. Criteria were solely selected by the working group if they related to the focus question and demonstrated a similar abstraction level. Moreover, all criteria had to be clearly defined and overlapping criteria were taken together.
- Sorting the criteria: the experts were asked to sort these criteria into piles on the basis of shared content or theme.
- Rating the relevance of the criteria: experts were asked to rate the perceived importance of the generated criteria on a 6-point scale (1 = not important, 6 = very important).

The concept mapping phase resulted in a number of clusters (criteria that were sorted onto the same pile most frequently by clinicians).

A meeting with experts was organized to operationalize the clusters. The overall content of the clusters could not be changed. During the meeting, the participants were given four tasks concerning each of the clusters.

- Examine the variables in the cluster. Which variables do you think should be discarded, or are there other variables that should be included?
- Each cluster should have a name that adequately describes the contents. Can you indicate an appropriate name for this cluster?
- To operationalize the cluster, it is necessary to ask the patient questions. What questions can be asked? Or what questionnaire(s) could be administered to assess how a patient scores on the cluster in question?
- What value should the cluster have for referral to a specific therapy?

On the basis of this process, criteria were added or omitted to the clusters. This meeting was followed by a conference call in which the clusters were operationalized and a first concept decision tool consisting of the clusters (the criteria on the tool) was presented.

### Phase 3: Validation study

The concept decision tool was filled out during the intake phase by the clinician and was composed of the criteria that were acquired from the concept mapping phase. One extra question was added to indicate whether the patient needed highly specialized care or not (yes/no). The clinicians based their answer to this question upon clinical impression. During the validation phase, the cut-off point of the final set of criteria was not shown to the therapists. At the end of the validation procedure, a meeting with the expert group was held to determine whether criteria that were not significantly associated with the clinical decision should be included in the final decision tool. A second meeting was organized to determine the cut-off score of the instrument.

### Participants

In total, 87 experts were approached to participate in the literature search and the concept mapping phase. Twenty-three of them provided search terms, 28 experts participated in the brainstorming session, 22 in the sorting task and 22 in the rating task. For concept mapping, our goal was to include a minimum of 15 experts to participate, since the average number of participants needed for reliable concept mapping is between 10 and 20 [18]. The data of five of the sorters could not be used due to incorrect execution of the sorting task. The pilot study included 20 therapists evaluating 44 patients, assessing the concept DTPD at the two mental health care institutions. Next, a larger validation study was performed in which seven centres participated, including 88 therapists evaluating 368 patients.

### Statistical analysis

Concept mapping software (Concept mapping, 2003) was used for the digital data collection process. Demographic data on the experts regarding sex, age, number of years of experience, title and setting, and the results of the statistical analysis were also collected. SPSS (IBM SPSS statistics, version 19.0.0) was used for the statistical analysis during concept mapping and Excel (Excel, 2010) for building a database during the validation study. R was used for modelling after the validation study.

Statistical analysis during concept mapping took the form of an analysis where criteria were grouped into clusters by putting the criteria into clusters that are more similar to one another and by determining the importance of the clusters (by ratings). These clusters were then operationalized and used as the final set of criteria in the validation phase. Statistical analysis in the this phase consisted of modelling the final set of criteria and determining a threshold for the set of criteria.

In the concept mapping phase, the frequency by which participants put criteria on the same pile was assessed (see Additional file 1 for more details about the analysis). These criteria were then plotted in a two dimensional plane. Criteria that were more similar (based on the frequency by which participants put the same criteria on the same pile) were closer to each other. A “goodness of fit” test that calculated the stress index (a goodness of fit measure) was conducted. Using cluster analysis, the criteria that were more similar to each other were grouped. The working group decided on the maximum and minimum number of groups (clusters). Subsequently, a stepped procedure was followed: starting at the maximum number of clusters, at each step two clusters were combined into one (hierarchical cluster analysis) until a minimum limit was reached. The working group based their decision not only on their clinical expertise (do certain criteria belong together in a cluster?) but also on the average number of clusters the participants had created, and on the bridging values. The bridging values are a measure of coherence between the criteria in the clusters (low means high coherence) and are an indication of the probability of experts sorting those criteria together into a single cluster. The mean value of rating on the 1–6 scale was calculated for each cluster and tested on significant differences to assess if the clusters should be weighed evenly. Reliability was subsequently evaluated by means of the point-biserial correlation, through which the correlation between individual sorting and group sorting was determined. The clusters acquired from concept mapping were considered to be the criteria on the decision tool.

For the validation study of this decision tool, similarity of the criteria with clinical decision was examined in a pilot study by calculating the percentage where a specific criterion was checked as positive and where at the same time clinicians indicated that this patient should be referred to highly specialized care. In the validation study, criterion validity was assessed. For criterion validity, the sensitivity and the specificity of the decision tool were evaluated. Sensitivity is the ability of the instrument to identify patients that belong to highly specialized care. Specificity is the ability to identify those patients that do not belong to highly specialized care. To determine whether patients do (or do not) belong to highly specialized care, clinical judgement was used. A multilevel model was applied as we expected that the clinical decisions within each treatment centre would correlate more than the clinical decisions between the centres. A binomial family of functions was used with a logit link function. The correlation structure was “exchangeable”. Using this model, sensitivity and 1-specificity were plotted in a Receiver Operating Characteristic Curve (ROC curve). Subsequently, the Area Under the Curve (AUC) was calculated. When sensitivity and specificity are both high, the AUC approaches 1. By using this model we could determine which of the criteria correlated significantly with clinical decision. Subsequently, easily implementable scoring systems were tested; the criteria were summed and sensitivity and specificity were determined at the specific cut-off points. For internal consistency, we calculated Cronbach’s Alpha.

## Results

### Phase 1: Literature search results

**Fig. 2:**
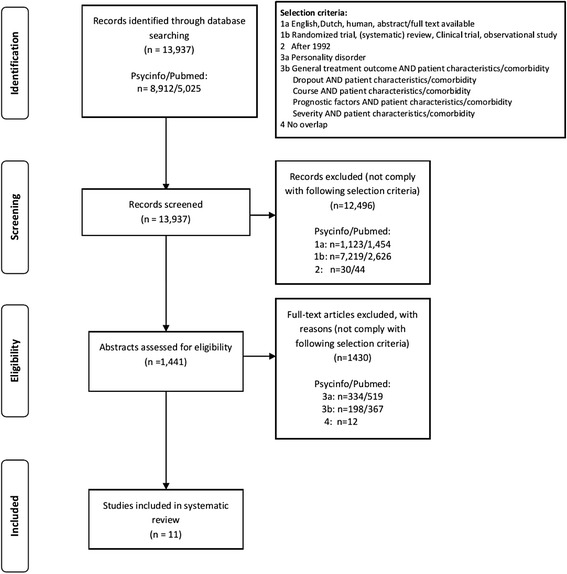
Flow diagram with studies selected for literature search

Respectively 8912 and 5025 studies were retrieved in PsycINFO and PubMed. These studies were selected according to the selection criteria (Fig. 2). The review yielded 11 studies, including four reviews and seven observational studies. Most of the studies considered patients with borderline personality disorders (BPD). Criteria found in the studies were mostly positively related to a specific treatment outcome or dropout, see Table 1. After removal of the duplicates, this resulted in 71 criteria, see Additional file 2. As none of these criteria were known to be directly related to referral of patients to highly specialized care, they were used in the brainstorm phase as input for the experts in formulating criteria for referral.

**Table 1:** Results of the literature review

Author	Type PS	Type of article	Criteria	Positive effect on
Barnicot, K. et al. (2011) [23]	BPD	Systematic review	Schizoid personality disorder High level of impulsivity Less pre-treatment suicidal behavior Lack of motivation to change Less internal, more external motivation to change Experiencing higher stigmatization Experiential avoidance Higher trait anxiety Higher anger level	Dropout
Barnow, S. et al. (2010) [26]	BPD	Review	Substance use disorders	Treatment outcome (suicidality/remission time)
Chiesa, M. et al. (2011) [27]	PD	Observational study	Deliberate self-harm	DSM-IV- (comorbidity)
Goodman, G. et al. (1998) [28]	BPD	Observational study	Initial depression and initial psychotic symptoms	Treatment outcome (SCID-P-comorbidity/ SCL-90R-symptom checklist)
Gunderson, J. G. et al. (2006) [22]	BPD	Observational study	Meet several criteria for obsessive-compulsive personality disorder Number of borderline personality disorder criteria Number of personality disorder criteria Number of axis-I disorders Early history of abuse and neglect Low GAF score Lower quality relationships	Treatment outcome (DSM-IV-Number of criteria/ lower GAF score)
McMurran, M. et al. (2010) [29]	PD	Systematic review	Lower age Lower level of educational attainment Lower-skilled occupation level Unemployed Convicted in court as an adolescent Parental divorce before the age of 10 Emotional neglect during childhood Less time alone Being in a relationship for less than six months Meet more than one PD diagnoses Meet more PD criteria Diagnosis of obsessive-compulsive PD, severe histrionic or antisocial PD and no specific PD Having a dependent PD Have a personality disorder in cluster A or B Higher level of narcism Higher level of impulsivity Fewer suicide attempts Higher trait anxiety Still be in the pre-consideration stage of change Less persistence Higher levels of avoidance Poor rational social problem-solving ability High level of carelessness in problem-solving High level of impulsivity in problem-solving More social competence Poor ego structure Less primitive defence Weaker adaptive defence style A greater denial of need for closeness Have conflicts regarding engagement and abandonment Fear of impulsive breakthrough of negative affect More externalizing defence Projective identification Lower level of general functioning Previous substance abuse Depressive self-image Less depressed No mood disorders Problems are focussed in one area	Dropout
Ryle, A. et al. (2000) [30]	BPD	Observational study	History of self-cutting Unemployed Alcohol abuse	Dropout
Skodol, A. E. et al. (2002) [31]	BPD	Review	Childhood sexual abuse Incest Lower age at first psychiatric contact Symptom chronicity Affective instability Magical thinking Aggression in relationships Impulsivity Substance abuse More Schizotypical features More Antisocial features More Paranoid features Number of borderline personality disorder criteria A greater number of axis II disorders Comorbidity of axis I and II disorders	Treatment outcome (DSM-IV: diagnostic criteria of borderline)
Thormählen B. et al. (2003) [32]	PD	Observational Study	Have a personality disorder in cluster A or B More distress Focus on 1 specific interpersonal problem Lower Age	Dropout
Yen, S. et. Al (2002) [33]	BPD, Schizotypical, Avoidant, and Obsessive Compulsive PD	Observational study	Measured number of physical attacks on another person in the past (with and without a weapon) More exposure to various types of trauma More lifelong PTSD Lower age at first traumatic experience	Severity (DSM-IV: more severe: Schizotypal, BPD; other types)
Yoshida, K. et al. (2006) [34]	BPD	Observational study	Overinvolvement in family relationships	Treatment outcome (lower GAF score)

### Phase 2: Concept mapping results

Twenty-seven experts completed questions about their demographics, see Table 2. The average age of the participants was 49 years, with on average 20 years of working experience. Most experts were psychiatrists working in an outpatient mental health care setting.

**Table 2:** Demographic variables

Demographic variables; concept mapping (*N* = 27)	
Sex (N(%) male)	15 (55%)
Mean age	48.85 (SD = 7.88)
Mean years of professional experience	20.37 (SD = 9.37)
Occupational setting (N(%))	
Nursing department	6 (22%)
Daycare	5 (19%)
Ambulatory mental health care institute	15 (56%)
Ambulatory private practice	1 (4%)
Discipline(N (%))	
Psychiatrist	18 (67%)
Psychotherapist/Clinical psychologist	6 (22%)
GZ psychologist	1 (4%)
Researcher	2 (7%)

#### Results of the brainstorm process

Following the brainstorming session, another 35 criteria were added to the criteria of the literature search. Selection of the criteria left a remaining total of 95 criteria, see Additional file 2 These criteria were included in the concept mapping system.

#### Results of the sorting process

The average number of clusters created by the participants during the sorting process was 10 (Range 5–23). The working group decided on a maximum number of clusters of 15 and a minimum of two with an optimal number of clusters of 6. In general, the bridging values (level of homogeneity) were low or acceptably low, which indicates a high homogeneity level for these six clusters. Cluster 4 exhibited the highest bridging value. The bridging values and the criteria in the clusters are shown in Additional file 3.

**Fig. 3:**
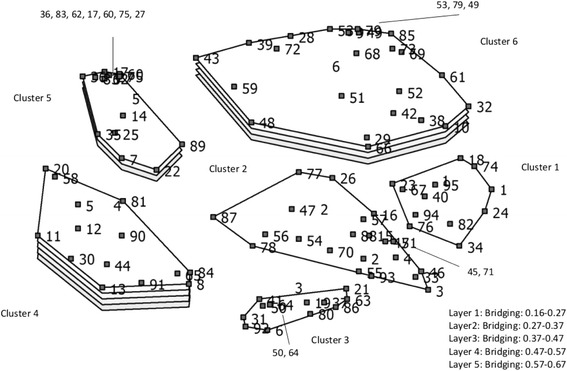
Cluster map with bridging scores

The clusters were presented in a cluster map with bridging values, see Fig. 3. This revealed that there were three clusters with very low bridging values (Cluster 1, Cluster 2, Cluster 3), indicating a high degree of cluster homogeneity. Goodness of fit was tested using the stress value (0 = very stable, 1 = distances between the criteria are completely at random). A stress value of 0.30 was found, which meant that the model fitted the data reasonably well.

#### Results of the rating process

The rating values per cluster are given in Additional file 3. Cluster 6 had a high rating score, despite its high bridging value. This means that, although the clinicians rated the criteria as important, the criteria often were not sorted into the same cluster (i.e. were not homogeneous). A t-test was performed to examine whether the scores from the rating task varied between the different clusters. As multiple t-tests were performed, a Bonferroni correction was applied (*p* < 0.003). Significant differences were found between cluster 1 and cluster 2. Also, a significant difference emerged between cluster 1 and cluster 3. This is caused by the higher rating of cluster 3 and cluster 2 as compared to cluster 1.

#### Results of the (final) expert meeting

Five experts attended the meeting, and three submitted input for the discussion in advance by email. During this meeting, the various clusters were defined that represented the content. They determined that six clusters that would be used. A relatively large number of variables were moved into cluster 6, which was in line with the high bridging values and corresponding low level of homogeneity in this cluster. Cluster 1, 2, 3, 4 and 6 were respectively operationalized as “Severe negative effect with disadaptive coping”, “Severe destructive behaviour to oneself or others”, “Multiple comorbid disorders on axis I and/or axis II due to severe psychiatric problems”, “Severe chronic traumatisation in childhood”, “Severe social and societal dysfunction: Global Assessment of Functioning (GAF)^2^ <45” and “Difficulties in developing a therapeutic relationship”. “Specialized treatment was not successful” was added. Also “Possibility and willingness to strictly follow minimal treatment conditions” was added as a starting point for assessing patients with the checklist. After the conference call, the set of criteria was finalized. All clusters were evenly weighted based on relevance. As there was not much difference in rating between the clusters, it was decided to weight them evenly. A preliminary cut off point was also chosen during the conference call (score ≥ 4).

#### Reliability

Reliability was estimated by correlating each individual sort matrix with the total matrix. The resulting correlations were all averaged. The reliability, if no Spearman-Brown correction was used, was 0.49.

### Phase 3: Validation study results

#### Pilot study

The similarities of the outcome of the criteria with clinical judgement were as follows: severe negative effect with disadaptive coping (77%), severe destructive behaviour to oneself and others (67%), multiple comorbid disorders on axis I and/or axis II due to severe psychiatric problems (72%), severe social and societal dysfunction: GAF < 45 (62%), severe chronic traumatisation in childhood (74%), difficulties in developing a therapeutic relationship (72%) and specialized treatment was not successful (81%). All criteria were highly similar. Severe social and societal dysfunction had the lowest similarity. Subsequently, we changed the cut-off score to GAF ≤ 50 because this yielded a higher similarity (68%).

#### Validation study

##### Demographics:

The characteristics of patients and therapists are shown in Table 3. There was no significant difference between the characteristics of the patients in the specialized and highly specialized care group. For therapists, only years of experience differed between the groups. In highly specialized care, therapists had more years of experience when compared to specialized care, see Table 3 (t(67.52) = 4.16, *p*-value = 9,2*10^-5).

**Table 3:** Characteristics patients/therapists divided into specialized/ highly specialized care

	highly specialized care (Patients: *N* = 110; Therapists: *N* = 29)	Specialized care (Patients: *N* = 268; Therapists: *N* = 59)
	Mean (sd)/percentage (%)	Mean (sd)/percentage (%)
Patients		
Age (years)	35,0 (11,7)	33,9 (10,6)
Gender (%men)	34% (35)	28% (64)
Therapists		
Age (years)	42.4 (11.2)	33,9 (10,4)
Experience therapist (number of years)	16.3 (8.7)**	8.7 (7,1)**
Talked to patient during intake (%Yes)	94.9%	100%

**p < 0.01

##### Model:

A multilevel model was applied. An overview of the outcomes in the model is showed in Table 4.

**Table 4:** Multilevel model

	Estimate	SE	*p*-value
Severe negative affect with disadaptive coping	2.530693	0.616013	3.99e–05**
Severe destructive behavior to oneself or others	0.917365	0.234275	9.01e–05*
Multiple comorbid disorders on axis I and/or axis II due to severe psychiatric problems	1.737646	0.849724	0.04086 *
Severe social and societal disfunction: GAF≤50	0.825936	0.380961	0.03016*
Severe chronic traumatisation in childhood	0.214238	0.807725	0.79083
Difficulties in developing a therapeutic relationship	-0.004092	0.265995	0.98773
Treatment in specialized care was not successful	1.208202	0.387603	0.00183*

*p<0.05, **p<0.01

##### ROC curve:

**Fig. 4:**
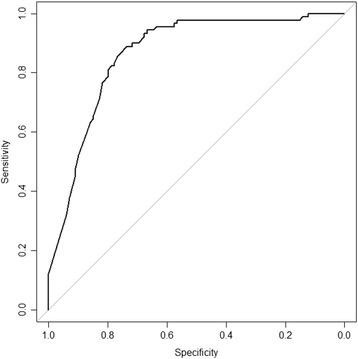
ROC curve with specificity on the x-axis and sensitivity on the y-axis

A ROC curve was plotted for the model, see Fig. 4. The area under the curve was high for the model, 0.865 (95% CI: 0.812–0.918) and subsequently the model discriminated well between low and high risk observations.

##### Cronbachs alpha:

Cronbachs alpha was 0.69.

##### Meeting:

During the meeting the experts agreed upon the criteria being part of the decision tool, as they cover expert opinion.

##### Scoring system:

In Table 5, the criteria were summed and sensitivity and specificity were determined at the specific cut-off points. A cut-off score of 4 and a cut-off score of 5 were associated with relatively good sensitivity and specificity, see Table 5.

**Table 5:** Number of criteria positively scored in relationship to sensitivity and specificity

Number of criteria positively scored	Sensitivity	Specificity
1. criteria or more	0.88	0.31
2. criteria or more	0.85	0.41
3. criteria or more	0.83	0.52
4. criteria or more	0.78	0.69
5. criteria or more	0.70	0.85
6. criteria or more	0.50	0.94
7. criteria	0.18	0.98

##### Meeting:

In the second meeting, the experts agreed that it was more important for the tool to be sensitive rather than specific, and subsequently a cut-off score of 4 was chosen. The decision tool was then finalized, see Table 6.

**Table 6:** Decision tool

Centre:		
Department:		
Name of professional/intaker		
Name of patient		
BSN number of patient		
	Yes/No	Value or finding
1. Severe negative affect with disadaptive coping	Yes	
	No	
2. Severe destructive behavior to oneself or others	Yes	
	No	
3. Multiple comorbid disorders on axis I and/or axis II due to severe psychiatric problems	Yes	
	No	
4. Severe social and societal disfunction: GAF≤50	Yes	
	No	
5. Severe chronic traumatisation in childhood	Yes	
	No	
6. Difficulties in developing a therapeutic relationship	Yes	
	No	
	NA^a^	
7. Treatment in specialized care was not successful	Yes	
	No	
	NA	
Number of times positively scored (=YES) Score ≥4?	Yes -> Go to question 8	
	No-> Not referred to highly specialized care based on this decision tool	
8. Possibility and motivation to conform to minimal treatment conditions for psychotherapy in intensive (day)care	Yes -> Referred to highly specialized care based on this decision tool	
	No-> Not referred to highly specialized care based on this decision tool	
	NA-> Referred to highly specialized care based on this decision tool	

^a^ *NA* Not applicable

## Discussion

Based on evidence from literature, a consensus method and a validation study a decision tool was developed to identify patients who may benefit from highly specialized care. Experts were consulted at every step to ensure good clinical relevance. The meetings ensured that the experts played a decisive role in the realization of the final result, while at the same time taking into account the generated clusters and ratings derived from the systematic concept mapping approach.

The DTPD consisted of seven criteria, as shown in Table 6. The criteria “Multiple comorbid disorders on axis I and/or axis II due to severe psychiatric problems”, “Severe social and societal dysfunction” and “Severe destructive behaviour to oneself or others” were similar to the criteria of “Comorbidity”, “Social functioning” and “Harm towards others” were found in the studies of Tyrer [7] and Crawford, Koldobsky, Mulder, and Tyrer [14]. As in the validation study, these criteria were significantly associated with clinical judgement. However, our decision tool also contained additional criteria that were considered important for clinical judgement, two of which were also significantly associated with clinical judgement. This may indicate that by using a systematic method, we covered a wider range of criteria compared to other studies.

### Limitations

Although a decision tool was developed that may cover a wide enough range of criteria to identify patients with personality disorders for highly specialized care, there are some limitations that need to be addressed. One limitation of the review was that an explicit statement containing information on the participants, interventions, comparisons, outcomes, and study design (PICOS) was not included. This approach was chosen to increase clarity, as the objective of this study was very broad (all interventions/comparisons were included and patients who were more severe and less severe were compared). Secondly, bias and quality of the studies was not assessed. All studies and subsequently all criteria on the decision tool that were found were included to minimize the risk of deleting important criteria. However, in the rating phase of concept mapping the importance of the criteria was assessed by the experts and criteria that were not relevant were excluded.

A limitation of the concept mapping methodology is that no specific combinations of criteria can be created in the concept mapping system. For example, when the combination of comorbid disorders and low functioning is considered to be important for referral to highly specialized care but the separate criteria are not, it was not possible to address this issue in the digital system. However, when relevant, combinations were discussed during the final meeting. In future studies, it might be feasible to define these combinations in a more structured manner and at an earlier phase by arranging a separate focus meeting or by using an additional consensus method for defining combinations (such as the Delphi method). Although the goal of the decision tool is to prevent ineffective treatment for patients with a personality disorder, “Treatment in specialized care was not successful” was a criterion of the tool. The reason behind this is that in reality many patients still have ineffective treatments. Additionally, the criterion was frequently mentioned by clinicians and rated as important. The concept mapping model fitted the data reasonably well (stress value was 0.30). According to Kane & Trochim [15] a value of between 0.20 and 0.35 implies a reasonable fit. This finding is underscored by a meta-analysis of concept mapping studies, in which 95% of the stress values ranged between 0.205 and 0.365 [19]. The reliability of our study was reasonably high, compared to the studies of Bedi [20] and Van Manen et al. [19] which found reliability estimates of respectively 0.45 and 0.56.

A limitation of the pilot and validation study is that clinical judgement was used as a gold standard. However up to date, there are no other validated questionnaires that can be used to measure the same construct. An additional limitation was that only one therapist provided input on both clinical judgement and on the criteria of the decision tool. This may have contributed to bias in the validation study and in future studies this should be addressed. As for the psychometrics, the interrater reliability was not assessed - and thus the degree of agreement between therapists is not known. In addition to this, the construct validity was not measured as we did not have any instrument which would measure the same construct. In future studies the interrater reliability should be assessed and when possible the construct validity. The internal consistency assessed by Cronbach’s alpha was relatively low. When criteria all measure one construct, Cronbach’s alpha would be high. However, a psychological construct consists of several different related aspects. When the construct is broader, as in the current study, more aspects are measured and the Cronbach’s alpha score will automatically be lower. In this way, a low alpha is not necessarily a disadvantage and may not prove a useful estimate. The selection of items on the instrument during the concept mapping phase ensured that only criteria that were thought to be clinically relevant by the experts were part of the the decision tool.

The results from the pilot study showed promise as the correlation between clinical judgement and judgement based on the set of criteria were high. The validation study confirmed the positive results for this study as the decision tool had high sensitivity and moderate specificity.

Although several forms of psychotherapy have proven to be effective in the treatment of personality disorders [21], not all patients profit from these treatments. Studies indicate that patients with more severe and complex personality disorders or specific characteristics may not profit from treatment [22] and are more prone to dropout [23]. Subsequently, they often have a long treatment history with negative results. There is, however, growing attention on early detection and early intervention to confine future damage caused by personality disorders [24]. The decision tool can be used in such a way as it may detect severe patients in an earlier stage of the disorder and improve their prognosis.

## Conclusion

In this study, we developed a decision tool to identify patients with personality disorders who may benefit from highly specialized care. This decision tool can be used by clinicians to identify patients who are in need of highly specialized treatment. Future research should focus on replication of this research in order to address the limitations in the current study and subsequently evaluate the long-term costs and quality of life of patients who are referred using the decision tool.

## Additional files

Additional file 1: Statistical analysis This part of the supplementary files provides detailed information about the analysis used in the concept mapping procedure. (DOCX 14 kb)

Additional file 2: Final set of criteria obtained via literature search or by brainstorming In this table, the final set of criteria that was extracted from the literature search and/or the brainstorm session is showed. (DOCX 18 kb)

Additional file 3: Cluster with average bridging and rating values. The table shows the criteria grouped by cluster and the average bridging values and rating values associated with the clusters. (DOCX 17 kb)

## Abbreviations

AUC: Area under the curve
DTPD: Decision tool personality disorder
GAF: Global assessment of functioning
PICOS: Patient, intervention, comparison, outcome, setting
PRISMA: Preferred reporting items for systematic reviews and meta-analyses
ROC: Receiver operating characteristic curve

## Footnotes

1 M.G (First Author) and D.S. (acknowledgements)

2 GAF = Global Assessment of Functioning scale [25]. It is a scale that is used by clinicians for rating social, occupational and psychological functioning of an individual.
