# Supplementary material for: Developing a decision tool to identify patients with personality disorders in need of highly specialized care
Source: BMC Psychiatry. 2017 Aug 31;17:317. doi: 10.1186/s12888-017-1460-6 (PMC5580206; doi:10.1186/s12888-017-1460-6)
Supplement: Supplementary file 1 — Statistical analysis This part of the supplementary files provides detailed information about the analysis used in the concept mapping procedure. (DOCX 14 kb) [file 12888_2017_1460_MOESM1_ESM.docx]

**Additional file 1 Statistical analysis**

A two-dimensional matrix, or ‘individual binary symmetric similarity matrix’ as this is known, was created for each participant. These individual sort matrices were then added together to obtain a ‘combined group similarity matrix’. Next, via non-metric multidimensional scaling (nDMS), all the points from the matrix were mapped, using an iterative approach, in a two-dimensional plot. The stress index is subsequently used as an index for the “goodness of fit” of the model (0=very stable, 1= distances are wholly random).

Clusters were created with the help of ‘agglomerative hierarchic cluster analysis’, in which use was made of Ward’s minimum variance algorithm.(Ward, 1963) First, the number of clusters was decided on, after which, at each stage in the analysis, two clusters were combined into one. Hence, first a decision had to be made about the maximum and minimum number of clusters, and the cut-off point for the number of clusters. Based on the bridging values and the average number of clusters, the working group respectively defined a minimum and a maximum number of clusters was defined, and the cut-off point. The bridging value is a measure of the coherence between the criteria in the clusters (0=high degree of homogeneity, 1 = low value of homogeneity). The clusters and the bridging values were then once again plotted in a two-dimensional map.

Subsequently, the rates of the clusters were examined on significant differences t-tests (with Bonferroni correction). The reliability was subsequently evaluated by means of the point-biseral correlation, through which the correlation between individual sorting and group sorting was determined.
