# Supplementary material for: Developing a decision tool to identify patients with personality disorders in need of highly specialized care
Source: BMC Psychiatry. 2017 Aug 31;17:317. doi: 10.1186/s12888-017-1460-6 (PMC5580206; doi:10.1186/s12888-017-1460-6)
Supplement: Supplementary file 2 — Final set of criteria obtained via literature search or by brainstorming In this table, the final set of criteria that was extracted from the literature search and/or the brainstorm session is showed. (DOCX 18 kb) [file 12888_2017_1460_MOESM2_ESM.docx]

**Additional file 2 Final set of criteria obtained via literature search or by brainstorming**

| Criterion | Literature search | Brainstorm additional |
| --- | --- | --- |
| A greater denial of the need for intimacy | ✓ |  |
| More affective instability | ✓ |  |
| Antisocial PD | ✓ |  |
| More magical thinking | ✓ |  |
| Low level educational attainment | ✓ |  |
| Comorbid Axis I, II and III disorders |  | ✓ |
| Lower age at first psychiatric contact | ✓ |  |
| Recent medical history shows numerous crisis admissions |  | ✓ |
| NO willingness to change |  | ✓ |
| Have conflicts regarding involvement and loneliness | ✓ |  |
| He/she costs society too much money |  | ✓ |
| Lower occupation level | ✓ |  |
| Lower level of general functioning | ✓ |  |
| A pathogenic home environment | ✓ |  |
| Poor ego structure | ✓ |  |
| Weaker adaptive defence style | ✓ |  |
| Incest | ✓ |  |
| Higher level of neurosis | ✓ |  |
| Psychotic symptoms | ✓ |  |
| Being in a relationship for less than 6 months | ✓ |  |
| More than one personality disorder | ✓ |  |
| Less psychotropic medication |  | ✓ |
| No specific PD | ✓ |  |
| Fear of sudden breakthrough of negative affect | ✓ |  |
| Lower age at first traumatic experience | ✓ |  |
| High level of carelessness in solving problems. | ✓ |  |
| Emotional neglect during childhood | ✓ |  |
| Investment in therapy is practically feasible |  | ✓ |
| High burden of suffering |  | ✓ |
| Unemployed | ✓ |  |
| Comorbidity complicated somatic suffering |  | ✓ |
| Low compliance | ✓ |  |
| Obsessive-compulsive personality disorder criteria | ✓ |  |
| Fear disposition | ✓ |  |
| Lower age | ✓ |  |
| Complications during pregnancy and childbirth |  | ✓ |
| Schizotypal comorbidity | ✓ |  |
| Less perseverance | ✓ |  |
| Inability to enter into a stable therapeutic relationship |  | ✓ |
| Mere externalizing defence | ✓ |  |
| Comorbid severe form of dissociative disorder |  | ✓ |
| Higher hostility level |  | ✓ |
| Tried in court as an adolescent | ✓ |  |
| Longstanding pattern of dysfunction |  | ✓ |
| High level of impulsivity | ✓ |  |
| Diagnosis of obsessive-compulsive PD | ✓ |  |
| Unclear diagnosis |  | ✓ |
| Experience higher stigmatization | ✓ |  |
| Low motivation, but some motivation to (be able to) comply with minimal treatment conditions |  | ✓ |
| Axis 1 comorbidity |  | ✓ |
| Aggression |  | ✓ |
| Sufficient capacity for change |  | ✓ |
| Less internal, more external motivation to change | ✓ |  |
| Crisis susceptibility |  | ✓ |
| Meet a higher number of PD criteria | ✓ |  |
| Higher level of symptom chronicity | ✓ |  |
| High level of impulsivity in solving problems | ✓ |  |
| Less time alone | ✓ |  |
| Measured number of physical attacks on another person in the past (with and without a weapon) | ✓ |  |
| Complex trauma in early childhood |  | ✓ |
| Investment in treatment possible as regards ego strength |  | ✓ |
| Separation from parents before age 10 | ✓ |  |
| Schizoid personality disorder | ✓ |  |
| Comorbid depression | ✓ |  |
| Higher number of lifetime para suicides |  | ✓ |
| Poor rational social problem-solving ability | ✓ |  |
| High anger level | ✓ |  |
| Urgent need for change |  | ✓ |
| Show willingness to change |  | ✓ |
| Suicidal tendencies |  | ✓ |
| Impulsivity | ✓ |  |
| Sufficient (minimal) adaptive capacity to function in a group or therapeutic environment |  | ✓ |
| Still be in the pre-consideration stage of change | ✓ |  |
| More avoidance based on experience | ✓ |  |
| More exposure to different types of trauma | ✓ |  |
| Evident problems in level of personality functioning, in personality organization. |  | ✓ |
| More aggression in relationships | ✓ |  |
| More antisocial comorbidity | ✓ |  |
| Lack of motivation to change | ✓ |  |
| Paranoid comorbidity | ✓ |  |
| A few isolated areas of health |  | ✓ |
| Projective identification | ✓ |  |
| Childhood sexual abuse | ✓ |  |
| Low GAF score with downward spiral | ✓ |  |
| NO willingness to change, but sufficient willingness |  | ✓ |
| Have a personality disorder in cluster A and B | ✓ |  |
| PTSD | ✓ |  |
| Deliberate self-harming | ✓ |  |
| Prior second echelon treatment yielded insufficient result. |  | ✓ |
| History shows more than one involuntary commitment |  | ✓ |
| Unable to move forward in several areas of life (work/school, social network and leisure activities) | ✓ |  |
| Comorbid addiction | ✓ |  |
| Severe histrionic PD | ✓ |  |
| Higher level of narcissism | ✓ |  |
| More avoidance | ✓ |  |
