# Supplementary material for: Developing a decision tool to identify patients with personality disorders in need of highly specialized care
Source: BMC Psychiatry. 2017 Aug 31;17:317. doi: 10.1186/s12888-017-1460-6 (PMC5580206; doi:10.1186/s12888-017-1460-6)
Supplement: Supplementary file 3 — Cluster with average bridging and rating values. The table shows the criteria grouped by cluster and the average bridging values and rating values associated with the clusters. (DOCX 17 kb) [file 12888_2017_1460_MOESM3_ESM.docx]

**Additional file 3 Cluster with average bridging and rating values**

| Clusters | Average Bridging value (SD) | Average rating value (SD) |
| --- | --- | --- |
| Cluster 1 | 0.25 (0.11) | 3.47 (0.47) |
| Higher level of neurosis | | |
| More avoidance based on experience | | |
| Fear of sudden breakthrough of negative affect | | |
| A greater denial of a need of intimacy | | |
| Higher level of narcissism | | |
| Evident problems in level of personality functioning, in personality organization. | | |
| High anger level | | |
| No specific PD | | |
| More externalizing defence | | |
| Projective identification | | |
| More avoidance | | |
| Fear disposition | | |
| Cluster 2 | 0.16 (0.14) | 3.9 (0.41) |
| Meet a higher number of PD criteria | | |
| More affective instability | | |
| Higher level of impulsivity | | |
| Impulsivity | | |
| Severe histrionic PD | | |
| More magical thinking | | |
| Diagnosis of obsessive-compulsive PD | | |
| Obsessive-compulsive personality disorder criteria | | |
| Antisocial PD | | |
| Deliberate self-harming | | |
| Suicidal tendencies | | |
| High level of impulsivity in problem-solving | | |
| Poor ego structure | | |
| Weaker adaptive defence style | | |
| Crisis susceptibility | | |
| High level of carelessness in problem-solving | | |
| Unclear diagnosis | | |
| More aggression in relationships | | |
| High level of symptom chronicity | | |
| PTSD | | |
| More antisocial comorbidity | | |
| Cluster 3 | 0.19 (0.09) | 4.05 (0.48) |
| Comorbid Axis I, II and III disorders | | |
| Axis I Comorbidity | | |
| Comorbid depression | | |
| Comorbid addiction | | |
| Comorbid severe form of dissociative disorder | | |
| Comorbidity complicated somatic suffering | | |
| More than one personality disorder | | |
| Paranoid comorbidity | | |
| Have a personality disorder in cluster A and B | | |
| Schizoid personality disorder | | |
| Psychotic symptoms | | |
| Schizotypal comorbidity | | |
| Cluster 4 | 0.67 (0.17) | 3.74 (0.97) |
| History shows more than one involuntary commitment | | |
| Unemployed | | |
| Lower occupation level | | |
| Low level of educational attainment | | |
| He/she costs society too much money | | |
| A few isolated areas of health | | |
| Less time alone | | |
| Being in a relationship for less than 6 months | | |
| Low GAF score with downward spiral | | |
| Recent medical history shows numerous crisis admissions | | |
| Higher number of lifetime para suicides | | |
| Longstanding pattern of dysfunction | | |
| Inability to move forward in several areas of life (work/school, social network and leisure activities) | | |
| Lower level of general functioning | | |
| Cluster 5 | 0.46 (0.19) | 3.73 (0.84) |
| Lower age at first traumatic experience | | |
| Previous second echelon treatment yielded insufficient result. | | |
| Lower age at first psychiatric contact | | |
| A pathogenic home environment | | |
| Lower age | | |
| Less psychotropic medication | | |
| Childhood sexual abuse | | |
| Complications during pregnancy and childbirth | | |
| Parental divorce before the age of 10 | | |
| Incest | | |
| Complex trauma in early childhood | | |
| More exposure to different types of trauma | | |
| Emotional neglect during childhood | | |
| Cluster 6 | 0.48 (0.15) | 3.83 (0.49) |
| No willingness to change, but sufficient willingness | | |
| Show willingness to change | | |
| Still be in the pre-consideration stage of change | | |
| Low motivation, but some motivation to (be able to)  comply with minimal treatment conditions | | |
|  |  |  |
| No willingness to change | | |
| Lack of motivation to change | | |
| Less internal, more external motivation to change | | |
| Urgent need for change | | |
| Experience higher stigmatization | | |
| Measured number of physical attacks on another person  in the past (with and without a weapon) | | |
| Sufficient (minimal) adaptive capacity to function in a  group or therapeutic environment | | |
|  |  |  |
| Investment in therapy is practically feasible | | |
| Tried in court as an adolescent | | |
| Inability to enter into a stable therapeutic relationship | | |
| Poor rational social problem-solving ability | | |
| High burden of suffering | | |
| Less perseverance  Higher hostility level  Aggression  Has conflicts regarding involvement and loneliness  Investment in treatment possible as regards ego strength  Sufficient capacity for change  Low compliance | | |
